# BrainNetVis: An Open-Access Tool to Effectively Quantify and Visualize Brain Networks

**DOI:** 10.1155/2011/747290

**Published:** 2011-03-14

**Authors:** Eleni G. Christodoulou, Vangelis Sakkalis, Vassilis Tsiaras, Ioannis G. Tollis

**Affiliations:** ^1^Institute of Computer Science (ICS), Foundation for Research and Technology—Hellas (FORTH), N. Plastira 100, GR-70013 Heraklion, Greece; ^2^Department of Computer Science, University of Crete, GR-71409 Heraklion, Greece

## Abstract

This paper presents BrainNetVis, a tool which serves brain network modelling and visualization, by providing both quantitative and qualitative network measures of brain interconnectivity. It emphasizes the needs that led to the creation of this tool by presenting similar works in the field and by describing how our tool contributes to the existing scenery. It also describes the methods used for the calculation of the graph metrics (global network metrics and vertex metrics), which carry the brain network information. To make the methods clear and understandable, we use an exemplar dataset throughout the paper, on which the calculations and the visualizations are performed. This dataset consists of an alcoholic and a control group of subjects.

## 1. Introduction

 One of the major issues in neuroscience is to describe how different brain areas communicate with each other during perception, cognition, and action as well as during spontaneous activity in the default or resting state. Mainly two different approaches for capturing and localizing brain activity motifs have been proposed; univariate spectrum based analysis and functional connectivity analysis [1]. Friston [2] defined functional connectivity as the statistical dependence between the activations of distinct and often well-separated neuronal populations.

Network models and graph theory provide a common framework for describing brain functional connectivity [3–5]. The interdependence between brain areas is estimated using multivariate neurophysiological signals (EEG, MEG, ECoG) and/or haemodynamic response images (fMRI). Then, a network is formed by corresponding either brain areas or channels to vertices and by considering an edge between two vertices if and only if the estimated interdependence is above a threshold. Regarding threshold selection, it is important to notice that it is a rather tricky part and there is currently no established way of favouring a specific threshold value. In practice, a broad range of threshold values is used to characterize the network. However, the authors propose two alternative approaches in selecting a threshold value based either on group statistics between specific graph-theoretic measures of the populations under analysis [6] or utilizing a signal-based technique of selecting the optimal visualization threshold using surrogate (artificially generated ensemble of data aiming at revealing the most significantly coupled brain regions) datasets to correctly identify the most significant correlation patterns [7]. The next step in the analysis, after edge identification, is to measure some networks statistics and characterize the network. Then, using the network characterization, one can draw conclusions on the effect of illnesses or of cognitive loads on functional connectivity [6–11].

In this study, we briefly refer to pairwise (bivariate) and multivariate interdependence measures, as well as linear and nonlinear ones, that have been successfully used as indices of cerebral engagement [12]. This information is important for the correct usage of the tool, especially for nonexpert users, as the application of these measures on the raw EEG data produces the input to our tool. The BrainNetVis tool provides a dynamic snapshot of the highly complex underlying neural mechanisms by means of graph visualization [13]. BrainNetVis is an open-access multiplatform tool, provided by ICS-FORTH, for graph representation and brain network visualization. Please note that BrainNetVis calculates the following presented metrics on the *synchronization matrices* (adjacency matrices) that the user should calculate in advance! However, the preprocessing section (Section3.2) briefly presents some widely used techniques to assess functional brain connectivity and form the adjacency matrix.

At this point, we refer to some already existing tools on the field. These tools capture different kinds of EEG information than BrainNetVis and they may be used complementary to it. One of them is EEGLAB [14], which we have been using extensively for better perception of the brain area. EEGLAB is an interactive Matlab toolbox for processing continuous and event-related EEG, MEG, and other electrophysiological data incorporating independent component analysis (ICA), time/frequency analysis, artifact rejection, event-related statistics, and several useful modes of visualization of the averaged and single-trial data. EEGlab offers also dipole localization functions. Some of the metrics that we implement have also been implemented in the Brain Connectivity toolbox (a matlab toolbox) by Rubinov and Sporns [15]. Other related toolboxes include MEA-Tools [16] and ERPWAVELAB [17]. In these toolboxes, however, the measures for quantifying channel interactions are mainly confined to the temporal crosscorrelation [16] and the coherence spectrum [17, 18]. However, more sophisticated interdependence techniques addressing not only linear but also nonlinear synchronization and causality are also available and applied in certain pathologies like Epilepsy [12]. Such measures can act complementary to graph theoretic indices that characterize brain networks as discussed in [19] and can be used as input to BrainNetVis.

The paper is organized as follows.  Section 2 presents essential information on the different ways of graph modelling and manipulations, using BrainNetVis.  Section 3 refers to the preprocessing needed (Section 3.2), the most commonly used menu calls and the GUI (Section 3.3), and the possible graph visualization options (Section 3.4). Our conclusion is given in Section 4.

## 2. Network Analysis

 Before presenting BrainNetVis, it is important to provide here some basic definitions from graph theory.

 A *graph G* = (*V*, *E*) defined on a set of *vertices V* = {*v*_1_,…, *v*_*n*_} and *edges E* = {*e*_1_,…, *e*_*m*_}, where each edge *e* ∈ *E* is an ordered or unordered pair of vertices. An ordered pair *e* = (*u*, *v*) ∈ *V* × *V* is called a *directed edge*, while an unordered pair *e* = {*u*, *v*}, where *u*, *v* ∈ *V*, is called an *undirected edge*. In case *u* = *v*, *e* is called a *self-loop*. In our study, we consider *simple* graphs, that is, graphs without self-loops. Also the cardinality of *V* is denoted by *n* (i.e., *n* = |*V*|).

A *weighted network G* = (*V*, *E*, *ω*) consists of a graph with vertex set *V* and edge set *E* augmented with an edge value function *ω* : *E* → ℝ that assigns to each edge *e* ∈ *E* a real value *ω*(*e*). Every weighted network *G* = (*V*, *E*, *ω*) corresponds to a real *n* × *n* matrix *W* = (*w*_*ij*_), *i*, *j* ∈ {1,2,…, *n*}, where *w*_*ij*_ is equal to value *ω*(*e*) of edge *e* = (*v*_*i*_, *v*_*j*_) if *e* ∈ *E*, or to 0 otherwise. If we reserve value 0 to mean the absence of an edge, then the correspondence between *G* and *W* is one to one. In this work, we consider a subset of weighted networks, which we call *synchronization networks*, where edge values are restricted to interval (0,1] and interpreted as strength of dependence between vertices.

In synchronization networks, higher edge values indicate stronger dependencies. To define the length of an edge, we should at least reverse the order of edge values by applying, for example, the inverse function *g* : (0,1]→[1, +*∞*), that is,

(1) $$\begin{array}{c} g\left(x\right)=\frac{1}{x} \end{array}.$$

We also propose another function *g* : (0,1]→[1, +*∞*), where

(2) $$\begin{array}{c} g\left(x\right)=1-{\log\;}_{2}\left(x\right) \end{array}.$$

These are definitions on how to transform the edge lengths in the case of synchronization networks. Which of the two functions performs better depends on the graph structure and on the metric or the visualization method that uses these functions. When choosing the appropriate formulation, one should consider that the function 1/*x* tends to +*∞* faster than the function 1 − log _2_(*x*) when *x* → 0^+^. Therefore, the edges with small values are assigned longer lengths with the 1/*x* function than those with the 1 − log _2_(*x*) function.

The length of a path from vertex *u* to vertex *v* is the sum of the lengths of the edges of the path. The shortest path distance from vertex *u* to vertex *v* is denoted by *d*_*G*_(*u*, *v*). If vertex *v* is unreachable from vertex *u*, then *d*_*G*_(*u*, *v*) = +*∞*.

## 3. Methods and Results

### 3.1. Exemplar Case

 In what follows, we are using the data of a specific use case, consisting of alcoholic and control subjects, in order to provide concrete examples of use of the application. Briefly, the specific study included 30 control subjects and 30 alcoholic subjects. Each subject was fitted with a 61-lead electrode cap (ECI, Electro-Cap International). All scalp electrodes were referred to *C*_*z*_. In this experiment, each subject was exposed to pictures of objects chosen from the 1980 Snodgrass and Vanderwart picture set [20]. The stimuli in each trial were randomized (but not repeated) and were presented on a white background for 300 ms at the center of a computer monitor. Their size was approximately 5–10 cm × 5–10 cm, thus subtending a visual angle of 0,05°–0,1°. Ten trials were shown, with the interval between trials fixed to 3.2 s. The participants were instructed to memorize the pictures in order to be able to identify them later. For each subject and for each trial and frequency band (0.5–4 Hz, 4–8 Hz, 8–13 Hz, 13–30 Hz, 30–45 Hz) the interdependence for each channel pair (there are 61 (61 − 1)/2 channel pairs since the number of active EEG channels is 61) was calculated using the coherence and the RIM methods. The results were stored in 61 × 61 interdependence matrices *W* with elements ranging from 0 to 1. The main finding of this study, using BrainNetVis, was that the alcoholic subjects have impaired synchronization of brain activity and loss of lateralization during the rehearsal process as compared to control subjects.

### 3.2. Preprocessing

 In order to create a graph, a matrix containing the EEG channel pairwise correlations is required. Thus, one needs to calculate the correlations among all pairs of electrodes and deduce the respective adjacency matrix, called *synchronization matrix*. There exist a number of measures that capture the linear and the nonlinear links between time-series in a frequency band in order to calculate the required correlations (in the EEG analysis context they are called synchronization indices). Three measures have been chosen after an extensive study in linear and nonlinear synchronization measures [12]: the typical magnitude squared coherence method (MSC) [21], a nonlinear bivariate measure for generalized synchronization (RIM) [22] and Partial Directed Coherence (PDC) [23]. The advantage of magnitude squared coherence is that it is well known and widely accepted. The advantage of RIM is that it is able to capture nonlinear patterns available in the signals, whereas PDC can measure causality.

**(1) Magnitude Squared Coherence (MSC)**

MSC (or simply coherence) has been a well-established and traditionally used tool to investigate the linear relation between two signals or EEG channels. Let us suppose that we have two simultaneously measured discrete time series *x*_*i*_ and *y*_*i*_, *i* = 1 … *N*. MSC is the cross-spectral density function *S*_*xy*_(*f*), which is simply derived via the fourier transform of the crosscorrelation, normalized by their individual autospectral density functions. Hence, MSC is calculated using the Welch's method as

(3) $$\begin{array}{c} \gamma_{xy}\left(f\right)=\frac{{\left|\langle S_{xy}\left(f\right)\rangle \right|}^{2}}{\left|\langle S_{xx}\left(f\right)\rangle \right|\left|\langle S_{yy}\left(f\right)\rangle \right|}, \end{array}$$

where 〈·〉 indicates window averaging. The estimated MSC for a given frequency *f* ranges between 0 (no coupling) and 1 (maximum linear interdependence).

**(2) A Robust Interdependence Measure (RIM)**

Given two scalar time series {*x*(*t*)}_*t*∈*𝕋*_ and {*y*(*t*)}_*t*∈*𝕋*_ with *𝕋* = {1,…, *N*}, which have been measured from dynamical systems *X* and *Y*, the dynamics of the systems are reconstructed using delay coordinates [24]

(4) $$\begin{array}{c} x\left(t\right)={\left[x\left(t\right),x\left(t+\tau \right),\ldots ,x\left(t+\left(m-1\right)\tau \right)\right]}^{T} \end{array}$$

and similarly we reconstruct **y**(*t*) from {*y*(*t*)}_*t*∈*𝕋*_, with an embedding dimension *m* and a delay time *τ* for *n* ∈ *𝕋*′ = {1,…, *N*′}, where *N*′ = *N* − (*m* − 1)*τ*. Regarding *τ* and *m*, they are parameters of Arnhold′s method [25]. Taken's [24] embedding theorems and their sequels (e.g., [26]) are existence proofs but they do not directly show how to get a suitable time delay *τ* or embedding dimension m from a finite time series. Empirical and heuristic criteria are employed for selecting *τ* and m. Usually, a choice of *τ* is the value for which the autocorrelation function first passes through zero, while m is determined using variations of false nearest neighbour statistics [27–29]. Parameter *τ* can also be calculated using the method of Fraser [30].

Let *r*_*t*,*j*_ and *s*_*t*,*j*_, *j* = 1,…, *k*, denote the time indices of the *k* nearest Euclidean neighbors of **x**(*t*) and **y**(*t*), respectively. Temporally correlated neighbors are excluded by means of a Theiler correction: |*r*_*t*,*j*_ − *t*| > *m* · *τ* and |*s*_*t*,*j*_ − *t* | > *m* · *τ*. For each *t* ∈ *𝕋*′, the average square distance of **y**(*t*) to all remaining points in {**y**(*j*)}_*j*∈*𝕋*′_ is given by

(5) $$\begin{array}{c} R_{t}\left(Y\right)=\frac{1}{N^{\prime }-1}\overset{N^{\prime }}{\underset{j=1,\;j\neq t}{\sum }}{\left|y\left(t\right)-y\left(j\right)\right|}^{2}. \end{array}$$

For each **y**_*t*_, the X-conditioned mean squared Euclidean distance is defined as

(6) $$\begin{array}{c} R_{t}^{\left(k\right)}\left(\frac{Y}{X}\right)=\frac{1}{k}\overset{k}{\underset{j=1}{\sum }}{\left|y\left(t\right)-y\left(r_{t,j}\right)\right|}^{2}. \end{array}$$

Quiroga et al. [25] defined the dependence measure

(7) $$\begin{array}{c} N\left(\frac{Y}{X}\right)=\frac{1}{N^{\prime }}\overset{N\prime }{\underset{t=1}{\sum }}\frac{R_{t}\left(Y\right)-R_{t}^{\left(k\right)}\left(Y/X\right)}{R_{t}\left(Y\right)}. \end{array}$$

The measure *N*(*X*/*Y*) is defined in complete analogy, and as interdependence measure between *X* and *Y*, we use the mean value (*N*(*X*/*Y*) + *N*(*Y*/*X*))/2.

**(3) Partial Directed Coherence (PDC)**

Let {**x**(*t*)}_*t*∈*ℕ*_ with **x**(*t*) = [*x*_1_(*t*),…,*x*_*n*_(*t*)]^*T*^ be a stationary *n*-dimensional time series with mean zero. Then, a vector autoregressive model of order *p* for **x** is given by

(8) $$\begin{array}{c} x\left(t\right)=\overset{p}{\underset{r=1}{\sum }}A\left(r\right)x\left(t-r\right)+ɛ\left(t\right), \end{array}$$

where **A**(*r*) are the *n* × *n* coefficient matrices of the model and *ɛ*(*t*) is a multivariate Gaussian white noise process with covariance matrix *𝚺*. In this model, the coefficients *A*_*ij*_(*r*) describe how the present values of *x*_*i*_ depend linearly on the past values of the components *x*_*j*_. In order to provide a frequency domain measure for Granger-causality, Baccala and Sameshima [23] introduced the concept of PDC. This measure is based on the Fourier transform of the coefficient series

(9) $$\begin{array}{c} \bar{A}\left(\omega \right)=I-\overset{p}{\underset{r=1}{\sum }}A\left(\omega \right)e^{-i\omega r}. \end{array}$$

More precisely, the PDC from *x*_*j*_ to *x*_*i*_ is defined as

(10) $$\begin{array}{c} \pi_{i\leftarrow j}\left(\omega \right)=\frac{\left|{\bar{A}}_{ij}\left(\omega \right)\right|}{\sqrt{\sum_{l=1}^{n}{\left|{\bar{A}}_{lj}\left(\omega \right)\right|}^{2}}}. \end{array}$$

The PDC *π*_*i*←*j*_(*ω*) takes values between 0 and 1 and vanishes for all frequencies *ω* if and only if the coefficients *A*_*ij*_(*r*) are zero for all *r* = 1,…, *p*.

The synchronization matrix created using one of the above methods serves as input to the BrainNetVis tool thus, it should be calculated separately and a priori. Please note that the presented tool currently implements only graph characterization measures and visualization schemes. It can be used with a variety of inputs in the form of the adjacency matrix. However, we provide the preprocessing section mostly for the interested but not expert user that wishes to investigate how graph analysis may be applied to the neuroscience field. In this sense, even if signal processing techniques are outside of the scope of the tool, we do describe the most widely used methods that provide the input for the further graph analysis. Nevertheless, it is true that most of the methods presented, linear (i.e., PDC) but mostly nonlinear ones (i.e., RIM), assume some kind of *stationarity*. Generally EEG distribution is considered as a multivariate Gaussian process even if the mean and covariance properties generally change from segment to segment. Therefore, strictly speaking, EEG meets quasistationarity because it can be considered stationary only within short intervals. Hence, the user should somehow test the stationarity assumptions prior to using these methods. Hopefully, a novel and prosperous technique capable of decomposing a multivariate time series into its stationary and nonstationary part is known as stationary subspace analysis (SSA) [31] and can be utilized to overcome the implicit stationarity constraints.

#### 3.2.1. Binary and Greyscale Networks on BrainNetVis

 BrainNetVis provides the option of using either a *binary* or a *greyscale* network by adjusting, respectively, the *Network Metrics Options* under the *View* drop down menu. In our use case, we provided as input to the tool a synchronization matrix describing the brain network of a *virtual* alcoholic patient. This virtual patient has been created by taking the means across the node and edge values over *all* 30 alcoholic subjects. We underline that this subject does not actually exist. We applied a binary network, using *threshold* = 0.4 and a greyscale network which we visualized using colormap scale. The edge length transformation function can also be selected under the same menu. We used

(11) $$\begin{array}{c} \ell \left(e\right)=\frac{1}{x}. \end{array}$$

**Figure 1:**
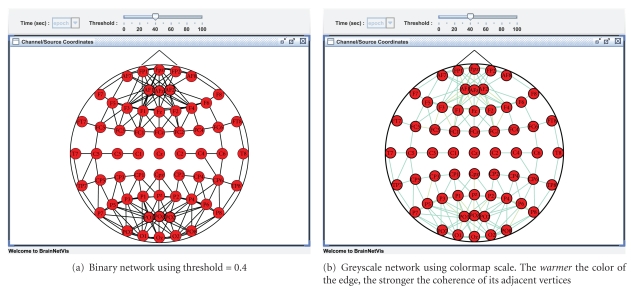
Example of weighted networks for a virtual alcoholic patient. Both pictures are produced with the Arnhold's method for broadband activity.

The results are depicted in Figure 1.

#### 3.2.2. Data Structure

 Two types of files are required for the algorithms that BrainNetVis encapsulates to run properly

1. A square synchronization matrix with the data from the EEG study (*required for the algorithms to function*).
2. A file containing a matrix of the labels and the coordinates of each electrode. The rows of the table correspond to the electrodes. The first column contains the electrodes' labels, and the other columns contain the coordinates of the electrodes. These will be either 2 columns (for 2D data, respective to *x* and *y* coordinates) or three columns (for 3D data, respective to *x*, *y*, and *z* coordinates). (*required for the visualization options*)

### 3.3. Menu Calls (GUI)

 The network metrics available in BrainNetVis will be presented here, in a way that follows the tool's structure.

#### 3.3.1. Global Network Metrics

 Networks are often classified into unifying categories in order to obtain a better understanding of their structure and function. *Network measures* are numbers which capture reduced information for graphs and describe essential properties. Network measures should catch the relevant and needed information, they should differentiate between certain classes of networks and be easily computed in order to be useful in algorithms and applications.

A very important global network metric is *clustering coefficient*. The clustering coefficient has been introduced by Watts and Strogatz [32] in 1998. For a vertex *v*, the clustering coefficient *c*(*v*) measures the connectivity of its direct neighborhood. The clustering coefficient *C*(*G*) of a graph is the average of *c*(*v*) taken over all vertices.

In the BrainNetVis application, we implement two different kinds of clustering coefficients, proposed by Zhang and Horvath (the first) and Onnela (the second). Zhang and Horvath proposed a definition which uses only the network values, in the context of gene coexpression networks. On the other hand, Onnela proposed a version of local clustering coefficient based on the concept of subgraph intensity, defined as the geometric average of subgraph edge values. Both metrics are defined in Table 1. It has to be noticed that the Onnela clustering coefficient definition suffers from the drawback that it requires an underlying binary network; if this is not available as a separate set of data, then presumably it must be obtained by discretizing the weighted edges.

The other important global network metric, included in the tool, is *assortative mixing*. This feature captures the similarity between properties of adjacent network vertices. Intuitively, this measure captures the tendency of network vertices to connect either to vertices with similar degrees (high degrees connected with high degrees and low degrees connected with low degrees) or to vertices that have dissimilar degrees (high degrees connected with low degrees). Newman [33] proposed an interesting measure to quantify the degree of similarity (dissimilarity) between adjacent vertices in a network using assortative mixing, which is given as the correlation between properties of every pairs of adjacent vertices. Each vertex may have assigned a single scalar, such as a centrality measure of the vertex position in a network, or a set of scalar properties. Then, the assortativity coefficient for an undirected graph is defined as the (sample) Pearson product-moment correlation coefficient. The formula of this computation is given in Table 1, and it is written in a symmetrical form. This equation can also be used for directed graphs by simply ignoring the direction of edges.

The value of the assortativity coefficient, *r*, lies in the range −1 ≤ *r* ≤ 1, with *r* = 1 indicating perfect assortativity and *r* = −1 indicating perfect disassortativity (perfect negative correlation between the properties of the vertices of the edges under consideration). Brain functional networks tend to be assortative [34, 35]. From computational studies, it has been observed that information gets easily transferred through assortative networks as compared to that in disassortative networks [36].

**Global network metrics on BrainNetVis**

**Figure 2:**
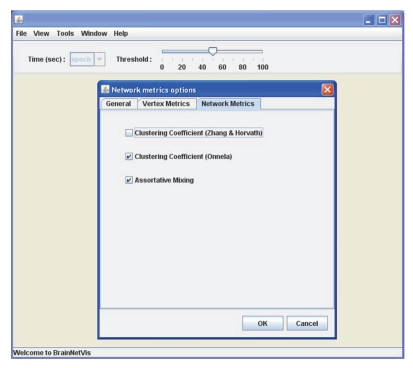
A menu screenshot depicting the selection of global network metrics.

BrainNetVis allows the calculation of the mentioned global network metrics by following the *Tools* menu (see Figure 2). Continuing the previous example on an alcoholic patient, we applied the simple *Clustering Coefficient* and the *Assortative Mixing*.

#### 3.3.2. Vertex Metrics-Centrality Measures

 The above concerned global network metrics. There exists a significant interest in local network properties as well, which concentrates on one node of interest. These properties are very important since at the local scale we can detect which vertices are the most relevant for the organization and functioning of a network. These local measures are commonly named *centrality measures* (or centrality indices) and have proved of great value in analysing the role played by individuals in social networks and in identifying essential proteins, keystone species, and functionally important brain regions.

**Centrality Measures Based on Neighbourhoods**

The simplest and most basic centrality measure is *degree centrality c*_*D*_(*v*) of a vertex *v*. In practice, this is the number of neighbours of the node of interest. In spite of the simplicity of this concept, degree is the most fundamental network measure and most other centrality measures are linked to it. The definitions of degree centrality, both for directed and for undirected networks are provided in Table 1.

In the case of greyscale networks, instead of using the term *degree centrality*, we use the term *strength centrality*. The formulas for strength centrality are defined correspondingly (Table 1). In BrainNetVis, strength centrality is presented as *normalized degree centrality*. This is accessed when the user chooses the *Normalized Metrics* on the Tools ⇒ Network Metrics Options ⇒ General tab and normalizes the edge values to range from 0 to 1 accordingly.

**Centrality Measures Based on Distances**

Another set of informative measures are the *Centrality Measures Based on Distances*, implying distances that information has to cover in order to be transferred through the network. The first metric that falls in this category is *closeness centrality*. Closeness can be regarded as a measure of how long it will take the information to spread from a given vertex to others in the network. Setting *G* = (*V*, *E*) as an undirected graph, the shortest path closeness centrality of vertex *v* ∈ *V* is defined as the inverse of the mean geodesic distance from vertex *v* to every other vertexe. A serious drawback of this metric is that it can only be used for connected graphs. A new measure, called *shortest path efficiency*, is proposed in Latora and Marchiori [37] and implemented in BrainNetVis application.

For a vertex *v*, Latora and Marchiori defined efficiency as

(12) $$\begin{array}{c} ef\left(v\right)=\frac{1}{n-1}\underset{u\neq v}{\sum }\frac{1}{d_{G}\left(v,u\right)}. \end{array}$$

The formula for that is provided in Table 1.

Note that (12) can also be used for disconnected graphs. If some vertices *v* and *u* are not connected, then they do not contribute to *ef*(*v*). In this case, *d*_*G*_(*v*, *u*) = +*∞*⇒1/*d*_*G*_(*v*, *u*) = 0. The global efficiency, *ef*(*G*), of a graph is the average of *ef*(*v*) taken over all vertices [37]

(13) $$\begin{array}{c} ef\left(G\right)=\frac{1}{n}\underset{v\in V}{\sum }ef\left(v\right)=\frac{1}{n\left(n-1\right)}\underset{v\in V}{\sum }\;\underset{u\neq v}{\sum }\frac{1}{d_{G}\left(v,u\right)}. \end{array}$$

In addition to *shortest path efficiency*, we are interested in *shortest-path betweenness centrality*. In this metric, two other nodes, apart from the central vertex *v*, are involved. We call these nodes *s* and *t*, respectively. This metric intuitively refers to the number of shortest paths which connect vertices *s* and *t* that pass through vertex *v*. In the formula provided in Table 1, the relative numbers *σ*_*st*_(*v*)/*σ*_*st*_ are interpreted as the extent to which vertex *v* controls the communication between vertices *s* and *t*. A vertex is considered central, if it is between many pairs of other vertices. Shortest-path betweenness centrality can be generalized to greyscale networks where the length of a path is equal to the sum of the lengths of its edges.

**Centrality measures based on Neighborhoods and on Distances in BrainNetVis**

**Figure 3:**
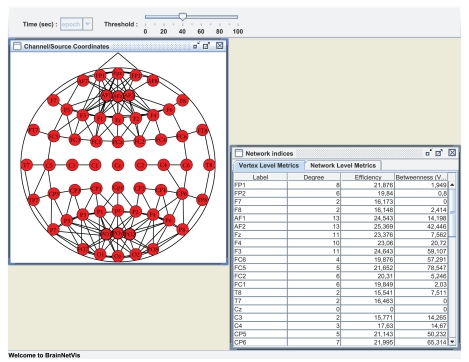
Centrality measures for the virtual alcoholic patient based on neighborhoods and on distances in BrainNetVis. The graph has been calculated by the Arnhold's method for broadband activity.

We applied the above types of centrality measures on our synchronization matrix of the alcoholic patient's EEG.  Figure 3 depicts the visualization of the individual's brain network using the *Static Visualization Method*. The *Binary Network* using threshold = 0.4 has been selected. The centrality measures calculated are the *Degree Centrality*, *Shortest Path Efficiency* and *Shortest Path Betweenness Centrality*. They are depicted on the respective table, shown in the same figure. Both the figure and the table with the metrics can be created by the following the *View* menu.

**Spectral Centrality Measures**

Another set of network metrics is based on the calculation of the *eigenvectors* of the adjacency matrix of the network, produced at the preprocessing step. Most of them are calculated by solving a linear equation system. These measures are called *Spectral Centrality Measures. Bonacich's eigenvector centrality* is one of them according to which the centrality of each vertex is proportional to the sum of the centralities of the vertices to which it is directly connected. The respective formula is presented in Table 1.

Expanding the simple Bonacich's eigenvector centrality, Hubbell [38] suggested yet another centrality measure based on the solution of a system of linear equations. *Hubbell's centrality* uses an approach based on directed weighted graphs where the weights of the edges may be real numbers. The general assumption of Hubbell's centrality is similar to the idea of Bonacich, but the centrality of a vertex depends both on its connection to other vertices and to exogenous input which sometimes is called boundary conditions. In this case, we include one more input to the equation *λ* **c** = *W* ^*T*^ **c** which describes Bonacich's eigenvector centrality. The result is shown on Table 1. This formula encapsulates the relative importance of endogenous versus exogenous factors in the determination of centrality.

The next spectral centrality measure, *subgraph centrality*, has been introduced by Estrada et al. [39]. It is calculated as the weighted sum of the number of closed walks in a graph, where longer walks receive lower weight than shorter ones. Very relative to the subgraphs of the network is the number of short walks of length *k*, starting and ending on vertex *v*_*i*_. This number is symbolized with *μ*_*k*_(*i*) on Table 1.

Last but not least, a very interesting idea was suggested by Demetrius et al. [40], describing *network entropy*. Evidence has been presented that this quantity is related to the capacity of the network to withstand random changes in the network structure. Network entropy is based on the Kolmogorov-Sinai (KS) entropy, which is a generalization of the Shannon entropy in that it describes the rate at which a stochastic process generates information. In our context, information corresponds to a sequence of vertices visited by an assumed Markov process on the network. Network entropy takes into account the impact of a vertex's removal on the network. This is captured by the product *π*_*i*_ *H*_*i*_ of the respective definition on Table 1. The interested reader could find more detailed information in [41].

**Spectral Centrality Measures in BrainNetVis**

We applied the above types of centrality measures on our synchronization matrix of the alcoholic patient's EEG. Using links from the *Tools* menu, we calculated the *Bonacich's Eigenvector Centrality*, *Hubbell's Centrality*, *Subgraph Centrality*, and *Network Entrophy*. One can define the type of networks with which he wishes to work (binary or greyscale) and also select the threshold value.

### 3.4. Graph Drawing Techniques

 Regarding the way in which the brain is depicted, BrainNetVis tool incorporates three different kinds of visualization as the follows.

#### 3.4.1. Static Visualization Method

 In this method, in order to visualize the topology of the emerged network, we create a static framework where each electrode is depicted by a node placed in a position similar to the actual electrode's position on the human cortex. Depending on the number of the electrodes of each experiment, an oval shape is outlined (which corresponds to the scalp) and inside this oval shape, a number *V* of circles exist that correspond to the electrodes placed on the subjects' head during the experiments.

#### 3.4.2. Multidimensional Scaling

**Figure 4:**
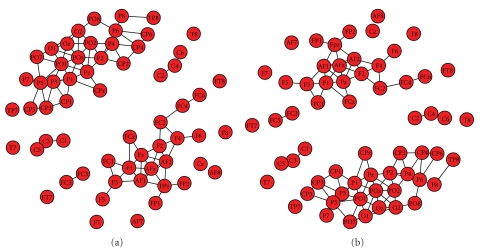
Multidimensional scaling.

 Multidimensional Scaling (MDS) is a family of techniques for analysis and visualization of complex data. The "beauty" of MDS is that we can analyze any kind of distance or similarity matrix, in addition to correlation matrices. Objects in a data set are represented as points in a geometric space; distance in this space represents proximity or similarity among objects. In our case, the objects are the electrodes and the distances among them are respective to their correlation in the synchronization matrix. In general, the goal of the analysis is to detect meaningful underlying connections among the electrodes which reflect the connections among different brain functional regions. In *BrainNetVis*, we incorporated a 2D visualization of the connections among electrodes. At this point, it has to be noticed that the more dimensions we use in order to reproduce the distance matrix, the better the fit of the reproduced is matrix to the observed matrix (i.e., the smaller the stress is). In fact, if we use as many dimensions as there are variables, then we can perfectly reproduce the observed distance matrix. Of course, our goal is to reduce the observed complexity of nature, that is, to explain the distance matrix in terms of fewer underlying dimensions. Some exemplar views of multidimensional scaling are shown in Figure 4

#### 3.4.3. Force-Based or Force-Directed Algorithms

**Figure 5:**
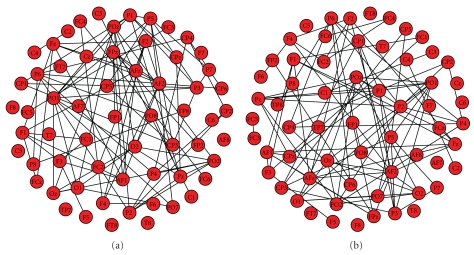
Binary stress.

 These are a class of algorithms for drawing graphs in an aesthetically pleasing way. Their purpose is to position the nodes of a graph in two-dimensional or three-dimensional space so that all the edges are of more or less equal length and there are as few crossing edges as possible. The force-directed algorithms achieve this by assigning forces amongst the set of edges and the set of nodes; the most straightforward method is to assign forces as if the edges were springs (see Hooke's law), and the nodes were electrically charged particles (see Coulomb's law). The entire graph is then simulated as if it were a physical system. The forces between its nodes change the dynamics and the layout of the system which at some point reaches its equilibrium state: at that moment, the graph is drawn. For force-directed graphs, it is also possible to employ mechanisms that search more directly for energy minima, either instead of or in conjunction with physical simulation. One of these mechanisms is binary stress (bStress), and it is the one we have incorporated in our tool. This model bridges the two most popular force directed approaches—the stress and the electrical-spring models—through the binary stress cost function, which is a carefully defined energy function with low descriptive complexity allowing fast computation via a Barnes-Hut scheme. Both electric-spring and stress approaches enjoy successful implementations and offer pleasing layouts to many graphs. Electric-spring models have the advantage of a lower descriptive complexity compared to the stress model. On the other hand, the stress function has a mild landscape, which allows utilizing powerful optimization techniques such as majorization. This way, good minima are usually achieved regardless of the initial positions. As far as the binary stress model is concerned, computationally, it is able to merge the advantages of both the electric-spring model and the stress model. Namely, it offers a low descriptive complexity, while at the same time, it is similar in its form to the known stress function, thus enabling the use of the majorization optimization scheme. More than other models, bStress emphasizes uniform spread of the nodes within a circular drawing area. In addition, bStress is suitable for drawing large graphs, not only because of its improved scalability, but also because it achieves good area utilization. Some exemplar views of binary stress visualization scaling are shown in Figure 5

More information on graph drawing techniques can be found in [13].

When we choose to visualize our graphs using the static visualization method, a change in the network metrics is not depicted on the output panel; this is because the electrode positions are stable and set from the beginning. Nevertheless, the changes in the calculations are saved in a matrix which is accessible by the end user. On the other hand, in multidimensional and binary stress modeling, the effects that take place when a network metric changes its value are depicted immediately after the change.

**Figure 6:**
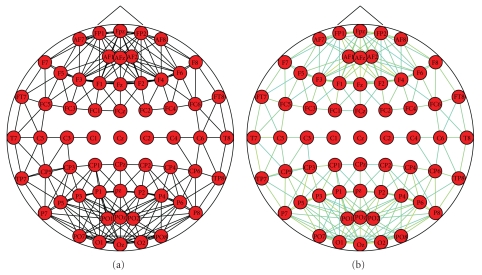
Static visualization for the synchronization matrix of the virtual control subject using (a) binary network and (b) greyscale network. Instead of scales of grey, the edge weights are depicted in colormap scale. Both pictures are produced with the Arnhold's method for broadband activity.

One can then set up the display options of his/her preference, for example, set up the way the graph vertices and edges will be displayed. As far as the nodes of the network are concerned, one can arrange their size, their color (*uniform* or *colormap*)and the depiction of the node labels. Regarding the edges, there exist three options for the color: *uniform* for directed networks, *greyscale* for greyscale networks (the intensity of the shadows of grey corresponds to the strength of the respective edge), and *colormap*. *Colormap* is also used in the case of greyscale networks but in this case colors are used: the closer the tint is to red color, the larger the strength of the respective edge is and the closer the tint is to blue color, the smaller the strength of the edge is. Moreover, one can adjust the size of the edge and whether this will be directed or not.  Figure 6 depicts the brain of the virtual control subject using both binary and colormap networks. In both cases, the threshold was set to 0.5.

## 4. Conclusion

 Using BrainNetVis, one can visualize and quantify the connections of the brain, based on EEG or MEG acquired signals. The inner brain connectivity is depicted as a graph; different sensor locations (electrodes) are visualized as nodes and their interconnections as edges. Therefore, scientists and clinicians will be able to get a better insight regarding brain connectivity and functionality and deduce more accurate results. We tested the tool using EEG data from alcoholic patients [7]. We were thus able to investigate some structural brain features that EEG and clinical data alone would not reveal. This tool can be easily used by the interested researcher, and it is accessible via http://www.ics.forth.gr/bmi/tools.html. It runs in every operating system that has JRE installed. Future work includes the support of the preprocessing methods mentioned in the same intuitive environment and the support of the binary European Data Format (EDF). Currently, simple ASCii text format is supported for simplicity and flexibility reasons.

## Figures and Tables

**Table 1:** Network and vertex metrics available in BrainNetVis.

Zhang and Horvath	$c_{Z}(v)=\sum_{i\neq j\in V\setminus \{v\}}{\hat{w}}_{vi}{\hat{w}}_{ij}{\hat{w}}_{jv}/\sum_{i\neq j\in V\setminus \{v\}}{\hat{w}}_{vi}{\hat{w}}_{jv}\Rightarrow$
	*c*_*Z*_(*v*) = (1/max _*i*,*j*_(*w*_*ij*_)) · (∑_*i*≠*j*∈*V*∖{*v*}_ *w*_*vi*_ *w*_*ij*_ *w*_*jv*_/∑_*i*≠*j*∈*V*∖{*v*}_ *w*_*vi*_ *w*_*jv*_)
	The weights have been normalized by max _*i*,*j*_(*w*_*ij*_).
	The above definition uses only the network values, in the context of gene coexpression networks.

Onnela	$c_{O}\left(v\right)=\left(1/\left(\begin{array}{c} \deg\;\left(v\right)\\ 2 \end{array}\right)\right)\sum_{i\neq j\in V\setminus \{v\}}{\left({\hat{w}}_{vi}{\hat{w}}_{ij}{\hat{w}}_{jv}\right)}^{1/3}\Rightarrow$
	$c_{O}\left(v\right)=\left(1/{\max\;}_{i,j}\left(w_{ij}\right)\left(\begin{array}{c} \deg\;\left(v\right)\\ 2 \end{array}\right)\right)\sum_{i\neq j\in V\setminus \{v\}}{\left(w_{vi}w_{ij}w_{jv}\right)}^{1/3}$
	Here, the edge values are normalized by the maximum value in the network,
	${\hat{w}}_{ij}=w_{ij}/{\max\;}_{l,k}w_{lk}$.

Assortative mixing	
Symmetrical weighted networks	*r* = (4*m*∑_{*u*,*v*}∈*E*_ *ρ*(*u*)*ρ*(*v*) − [∑_{*u*,*v*}∈*E*_(*ρ*(*u*)+*ρ*(*v*))]^2^)/(2*m*∑_{*u*,*v*}∈*E*_(*ρ*(*u*)^2^ + *ρ*(*v*)^2^) − [∑_{*u*,*v*}∈*E*_(*ρ*(*u*)+*ρ*(*v*))]^2^)
Directed weighted networks	$r=\left(H\sum_{(u,v)\in E}\omega (u,v)\rho (u)\rho (v)-AB\right)/\left(\sqrt{H\sum_{(u,v)\in E}\omega \left(u,v\right)\rho {\left(u\right)}^{2}-A^{2}}\sqrt{H\sum_{(u,v)\in E}\omega \left(u,v\right)\rho {\left(v\right)}^{2}-B^{2}}\right)$
	*A* = ∑_(*u*,*v*)∈*E*_ *ω*(*u*, *v*)*ρ*(*u*)
	*B* = ∑_(*u*,*v*)∈*E*_ *ω*(*u*, *v*)*ρ*(*v*)
	*H* = ∑_*e*∈*E*_ *ω*(*e*) is the sum of all values of edges in *E*.

Degree centrality *c*_*D*_(*v*) of vertex *v *	
Undirected binary network	Degree deg (*v*) of vertex *v*
Directed binary network	In-degree *c*_*iD*_(*v*) = deg ^−^(*v*)
	Out-degree *c*_*oD*_(*v*) = deg ^+^(*v*)

Strength centrality *c*_*S*_(*v*)	
Greyscale symmetric network	Strength *s*(*v*) of vertex *v*
Greyscale assymetric network	In-strength: *c*_*iS*_(*v*) = *s* ^−^(*v*)
	Out-strength: *c*_*oS*_(*v*) = *s* ^+^(*v*)

Shortest-path Efficiency	*c*_*Ef*_(*v*) = (1/*n*_*Ef*_)∑_*u*≠*v*_1/*d*_*G*_(*v*, *u*), where *n*_*Ef*_ = *n* − 1

Shortest-path Betweeness centrality *c*_*B*_(*v*) of a vertex *v* ∈ *V*	*c*_*B*_(*v*) = (1/*n*_*B*_)∑_*s*∈*V*∖{*v*}_∑_*t*∈*V*∖{*v*,*s*}_(*σ*_*st*_(*v*)/*σ*_*st*_), where *σ*_*st*_ is the number of shortest (*s*, *t*)-paths
	*σ*_*st*_(*v*) is the number of shortest (*s*, *t*)-paths passing through some vertex *v* other than *s*, *t* and *n*_*B*_ = (*n* − 1)(*n* − 2) is a normalizing constant.

Bonacich's eigenvector centrality	*λc*(*v*_*i*_) = ∑_*j*=1_^*n*^ *w*_*ji*_ *c*(*v*_*j*_)
	In matrix notation with **c** = [*c*(*v*_1_),*c*(*v*_2_),…,*c*(*v*_*n*_)]^*T*^, this yields:
	*λ * **c** = *W* ^*T*^ **c**.
	This type of equation is well known and solved by the eigenvalues and eigenvectors of *W* ^*T*^.
	We call the eigenvector **s** = [*s*_1_,…,*s*_*n*_]^*T*^ of the maximal eigenvalue of *λ * **c** = *W* ^*T*^ **c** principal eigenvector. Then, the eigenvector centrality of node *v*_*i*_ is defined as: *c*_*EV*_(*v*_*i*_) = |*s*_*i*_|/||**s**||_*p*_,
	where the centrality vector **s** is normalized by dividing it by its *p*-norm
	||**s**||_*p*_ = (∑_*i*=1_^*n*^|*s*_*i*_|^*p*^)^1/*p*^ 1 ≤ *p* < *∞*, and ||**s**||_*p*_ = max _*i*=1,…,*n*_{|*s*_*i*_|} *p* = *∞* to produce centrality scores *c*(*v*_*i*_) ≤ 1.

Hubbell's centrality	**c** = *αW* ^*T*^ **c** + **e** where **c** = [*c*(*v*_1_),*c*(*v*_2_),…,*c*(*v*_*n*_)]^*T*^ and **e** = [*e*_1_,*e*_2_…,*e*_*n*_]^*T*^.
	In order to get meaningful results, *α* should be chosen according to restriction |*α*| < 1/*λ*_1_, where *λ*_1_ is the maximum value of an eigenvalue of *W*.
	This restriction is not mentioned in the literature.

Subgraph centrality of vertex *v*_*i*_	It is given by the *i*th diagonal entry of the *k*th power of the adjacency matrix, *A*
	*c*_*SG*_(*v*_*i*_) = ∑_*k*=0_^*∞*^ *μ*_*k*_(*i*)/*k*! with number of closed walks: *μ*_*k*_(*i*) = (*A* ^*k*^)_*ii*_.
	This measure generalizes to greyscale networks by substituting matrix *W* for *A*.
Network entropy	$H(\hat{P})=-\sum_{i,j}^{}\pi_{i}{\hat{p}}_{ij}\log\;\;{\hat{p}}_{ij}=\sum_{i}^{}\pi_{i}H_{i}$
	To produce the above equation, we have set a Markov matrix *P* = [*p*_*ij*_] be the stochastic process which defines the information source and its stationary distribution *π* : *πP* = *π*.

## Appendix

We present here a summary of the metrics used at BrainNetVis and their placement under the tools menu. The main menu when the GUI opens contains the options: *File, View, Tools, Window, and Help. *

File This drop-down menu includes the following tabs.

- *Import*. Following this tab, the user can give as input the greyscale matrix that corresponds to the network of interest along with the vertex coordinates. He can browse his computer for these required files.
- *Export*. It is used to export the produced visualizations to a file with various formats (.eps,.pdf,.jpg, etc)
- *Exit*. It is used to quit the GUI.
- *Output*. One can export all the metrics of the examined network at a.*txt* file, which is saved in the same directory with the tool executable.

View Under the *View* drop-down menu, one can find the following.

- *Network Visualization*. One can choose among the three supported visualization techniques: Channel/Source coordinates, Multidimensional Scaling and Binary Stress, described in detail in Section 3.4
- *Network Metrics*. Following this tab, the user can ask either for the *Vertex level metrics* table, which contains the values of the vertex metrics that interest the user (and which he chooses under the *Tools* drop-down menu), or for the *Network level metrics*, which contains the values of the global network metrics.

Tools This menu contains the following.

- *Display Options*. Following this tab, the user can set up the display of the graphs. He can set his preferences concerning the nodes (size, color, label, font) and/or the edges (size, color, direction, arrow size).
- *Network Metrics Options*. Three tabs appear in this sub-menu. The first one is named *General* and contains options like if the network is directed or not, binary or not and synchronization network or not. In the latter case, the tool provides an option on the normalization of the edge length. The second tab is named *Vertex Metrics* and contains options for all the vertex metrics described in Section 3.3.2. Finally, the last tab is named *Network Metrics* and contains options for the network metrics described in Section 3.3.1.

Window Here, the user can change the size of the window of the GUI.
